# Hepatitis B Virus Induces Cell Proliferation via HBx-Induced microRNA-21 in Hepatocellular Carcinoma by Targeting Programmed Cell Death Protein4 (PDCD4) and Phosphatase and Tensin Homologue (PTEN)

**DOI:** 10.1371/journal.pone.0091745

**Published:** 2014-03-14

**Authors:** Preeti Damania, Bijoya Sen, Sadaf Bashir Dar, Satendra Kumar, Anupama Kumari, Ekta Gupta, Shiv Kumar Sarin, Senthil Kumar Venugopal

**Affiliations:** 1 Institute of Liver and Biliary Sciences, New Delhi, India; 2 South Asian University, New Delhi, India; The University of Hong Kong, China

## Abstract

Hepatitis B viral infection-induced hepatocellular carcinoma is one of the major problems in the developing countries. One of the HBV proteins, HBx, modulates the host cell machinery via several mechanisms. In this study we hypothesized that HBV enhances cell proliferation via HBx-induced microRNA-21 in hepatocellular carcinoma. HBx gene was over-expressed, and miRNA-21 expression and cell proliferation were measured in Huh 7 and Hep G2 cells. miRNA-21 was over-expressed in these cells, cell proliferation and the target proteins were analyzed. To confirm the role of miRNA-21 in HBx-induced proliferation, Hep G 2.2.1.5 cells (a cell line that expresses HBV stably) were used for miRNA-21 inhibition studies. HBx over-expression enhanced proliferation (3.7- and 4.5-fold increase; n = 3; p<0.01) and miRNA-21 expression (24- and 36-fold increase, normalized with 5S rRNA; p<0.001) in Huh 7 and Hep G2 cells respectively. HBx also resulted in the inhibition of miRNA-21 target proteins, PDCD4 and PTEN. miRNA-21 resulted in a significant increase in proliferation (2- and 2.3-fold increase over control cells; p<0.05 in Huh 7 and Hep G2 cells respectively) and decreased target proteins, PDCD4 and PTEN expression. Anti-miR-21 resulted in a significant decrease in proliferation (p<0.05) and increased miRNA-21 target protein expression. We conclude that HBV infection enhances cell proliferation, at least in part, via HBx-induced miRNA-21 expression during hepatocellular carcinoma progression.

## Introduction

Hepatocellular carcinoma (HCC) is one of the major malignant neoplasm, affecting more than half a million people worldwide each year, and has a multifactorial etiology including hepatitis B or hepatitis C infections and alcoholism [1]. Several studies have shown that there is an association between hepatitis B infection and HCC, but precise molecular mechanisms that regulate the proliferation in these cells remain unknown [1], [2].

Several studies have shown that the ‘X’ open reading frame of Hepatitis B oncogenic viral genome, encoding a 17 kD protein, is necessary for in vivo infection and stimulates the HBV replication by increasing mitochondrial calcium uptake as well as a critical contributor in the initiation of neoplastic transformation [2]–[4]. Paterlini et al. found that HBV DNA was integrated in the chromosomal DNA of hepatocytes in HBV-related HCC patients negative for HBV surface antigen (HBsAg), but positive for X-transcript, implying a significant role of the integrated-X gene in transformation [5]. HBx knock-in transgenic mice at the p21 locus developed liver tumor at 18 months after birth, suggesting the oncogenic potential of X protein [6].

Several studies reveal that HBx can promote proliferation, motility and invasion in human hepatocytes by up-regulating MEKK2, MIG, MMP-9, IKKα and Capn4 [7]–[11]. It is also suggested that HBx may possibly alter the adhesion-de-adhesion balance of the cells in the primary tumor site, favoring integrin-mediated cell migration as well as modulate cell cycle regulatory proteins of G1 phase in a calcium-dependent manner [12], [13]. Emerging data show that miRNAs are involved in HBx-induced cell proliferation and invasion in HCC [14]–[18].

microRNAs (miRNAs) are endogenous, non-coding ∼22 nucleotide RNA molecules, shown to modulate gene expression via post-transcriptional manner, thus becoming crucial regulators in complex gene regulatory networks. Several studies are available to show the deregulated expression of miRNAs in cancer and reveal a key role in the initiation and progression of the disease [19]. It was shown that at least 17 miRNAs were down-regulated in HCC, which in turn activated many oncogenic pathways including cell cycle progression, while 6 miRNAs were up-regulated, which were responsible for the anti-tumor immune response [20]. Recently it was shown that miRNA-148a is down regulated by HBx and caused increased tumorigenesis [14]. In another study, HBx up-regulated miRNA-143, thereby promoting metastasis [21]. Very limited data is available on the role of HBx-induced miRNAs in HCC.

MicroRNA-21 (miRNA-21) is a multifaceted microRNA, regulating multiple genes involved in several cellular programs. APAF1, the core of the apoptosome, essential for activating caspases to initiate apoptosis, contains a miRNA-21 target site in its 3′UTR, and is found to be down-regulated while miRNA-21 is up regulated in gliomas, augmenting proliferation [22]. MiRNA-21 induces AP-1 activity in response to ras onco-protein by directly repressing tumor-suppressor gene PDCD4, contributing to tumorigenesis by auto-regulatory mechanism [23]. In anaplastic thyroid carcinoma and lung cancer, early activation of ras and its two downstream pathways raf-MAPK and PI3K pathways triggered high miRNA-21 expression in neoplastic transformation in vivo [24]. It has been reported that miRNA-21 plays a vital role in keratinocyte migration and in re-epithelialization during wound healing, directly targeting TIMP3 in vitro and in vivo [25]. By down-regulating PDCD4, miRNA-21 contributes to glioblastoma proliferation while its inhibition induced apoptosis and decreased cell cycle progression, down-regulating EGFR, activated akt, cyclin D and bcl-2 in vitro and in vivo suggesting an important therapeutic potential of miRNA-21 [26], [27]. Thus, the fact that miRNA-21 is frequently elevated in most malignancies and its in vivo knockdown suppresses tumorous potential, suggests that its high levels are essential for promoting pathological cell growth. In HCC tissues and multiple HCC cell lines, it promoted cell proliferation, invasion and migration by repressing the expression of tumour-suppressor genes Programmed Cell Death Protein-4 (PDCD4) and Phosphatase and Tensin homologue (PTEN) [28], [29].

HBx and miRNA-21, both are reported to play a causal role in the proliferation and neoplastic transformation. However, it is not known whether HBx mediates the proliferation through miRNA-21. Hence, in this study we investigated the role of miRNA-21 in HBx-induced proliferation in hepatoma cells.

## Materials and Methods

### Cell Culture and Transfection

Human hepatoma cell lines HepG2, Huh7 and HepG2.2.15 were maintained in Dulbecco's modified Eagle's medium (Hi Media, India) supplemented with 10% fetal bovine serum (Invitrogen, USA) containing penicillin-streptomycin antibiotics (Invitrogen, USA) at 37°C in a humidified atmosphere with 5% CO_2_.

For transfection with plasmid DNA, cells were plated in 6-well plates at a density of 1×10^6^ cells/well. After 24 hours, the transfection was performed using lipofectamine LTX with PLUS reagent (Invitrogen) according to the manufacturer's instructions. For transfection with pre-miRNA oligos (Sigma Aldrich, USA) or anti-miR-21, siPORT NeoFX transfection agent (Applied Biosystems, USA) was used according to the manufacturer's instructions. pSG5-HBx plasmid was used to transfect HBx gene in hepatic cells, and as a control, an empty vector was used in all the plasmid transfection experiments. To test the efficiency of transfection, enhanced Green Fluorescent Protein-N1 (eGFP-N1) plasmid vector was used in initial experiments. For inhibition of miRNA-21, anti-miR-21 oligos were transfected same as described above followed by transfection of HBx plasmid after 24 hours of anti-miR-21 transfection. After 48 hours of transfection of HBx plasmids, the cells were collected for the Western blot experiments. Cell lysates were collected for running the western blots and total RNA isolation. All transfection experiments were done in duplicates and repeated at least three times.

### RNA Isolation, quantification and reverse transcription PCR analysis (qPCR)

Total RNA enriched with miRNAs was isolated using mirVana miRNA Isolation Kit (Ambion, USA) as described by us previously [30], [31]. To evaluate miRNA-21 expression, real-time quantitative reverse transcriptase-polymerase chain reaction was performed. cDNA was generated by reverse transcription using 10 ng of total RNA according to the manufacturer's instructions (Universal cDNA synthesis kit, Exiqon, Denmark). Following first strand cDNA synthesis, SYBR green qPCR was carried out using miRCURY LNA Universal RT microRNA PCR in a LightCycler 480 Real-Time PCR System (Roche, India) to quantify relative miRNA-21 levels which were normalized using control 5S RNA (Exiqon). The relative expression was analyzed using the 2^ΔΔCT^-method [32].

### Western Blot Analysis

Cells grown in 6-well plates were washed twice with phosphate buffered saline, followed by lysis using mammalian protein extraction reagent and HALT protease inhibitor cocktail (Thermo Scientific, USA). The protein content was quantified using bicinconinic acid reagent (Thermo Scientific, USA). The protein samples (30–60 µg/lane) were separated using 10% polyacrylamide gels and transferred to polyvinylidene fluoride membranes. Membranes were blocked with 5% non-fat dry milk in tris buffered saline (TBS) and proteins were detected using antibodies against HBx (provided by Dr Vijay Kumar, ICGEB, New Delhi), PTEN, PDCD4, Akt or Phospho-Akt (Cell Signalling Technology, USA). Anti-β-actin was used as an internal control (Santa Cruz Biotechnologies, USA). Membranes were incubated with the horseradish–peroxidase conjugated secondary antibodies, and the blots were visualized using enhanced chemi-luminescence kit (Amersham, Germany), followed by developing on the films.

### Cell Proliferation Assay

The cells were seeded in 96 well plates at a density of 8,000 cells/well and were transfected with plasmid DNA or miRNAs in separate experiments. After 48–72 hours, they were assayed for proliferation using WST-1 reagent (Roche) as described previously [30].

### Statistical Analysis

All the experiments were performed at least three times. The data were expressed as mean ± standard deviation and p<0.05 was considered as statistically significant. The statistical significance was calculated using analysis of variance, followed by paired t-tests.

## Results

**Figure 1:**
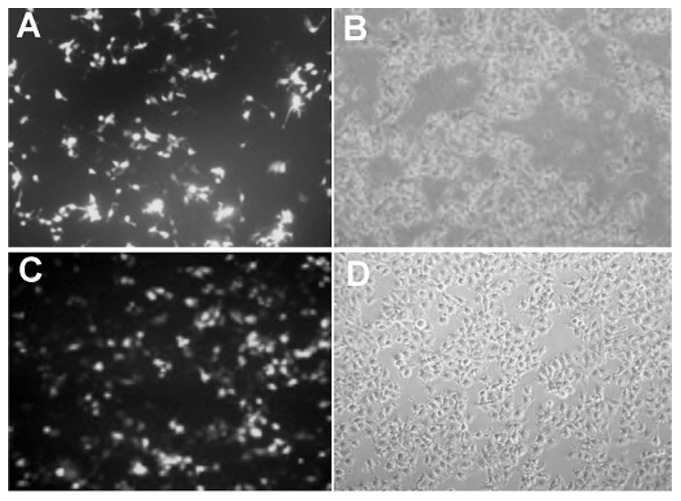
Transfection efficiency in Huh 7 and Hep G2 cells. Both Huh 7 (A and B) and Hep G2 (C and D) cells were transfected with eGFP-N1 plasmid using the similar transfection conditions as HBx. After 48 hours of transfection, the cells were observed (10× magnification). Both A and C are fluorescent pictures and B and D are corresponding phase-contrast pictures.

**Figure 2:**
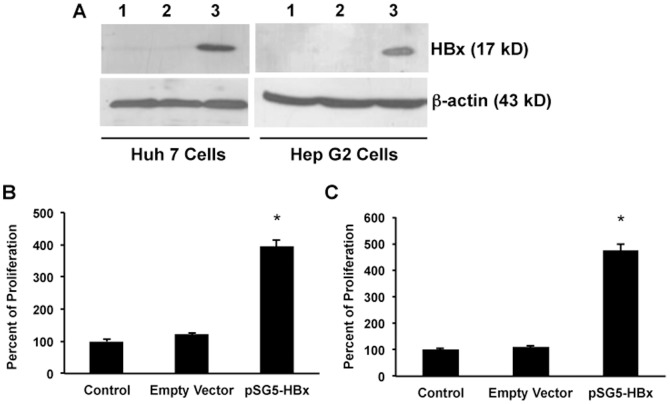
Effect of HBx on the proliferation. HBx protein was over-expressed in both Huh 7 and Hep G2 cells (A). HBx was over-expressed in hepatoma cells and the empty vector was used as transfection control. Lane 1, Control; lane 2, Empty vector; and lane 3, HBx transfected cells. Cell proliferation assay was performed using WST1 reagent in Huh 7 (B) and Hep G2 (C) cells. After 48 hours of transfection the proliferation assay was performed. (n = 3; *p<0.01).

MiRNA-21 has been shown to induce cell proliferation in a variety of cells. Previous studies have shown that HBx induces cell proliferation of the HCC cells [7], [13], [24]. Although, HBx induces proliferation via several mechanisms, very limited data is available on the role of HBx-induced miRNAs in regulating the proliferation and metastasis in HCC [9], [14], [18]. Hence, in this study we have tested the hypothesis that HBx might induce cell proliferation, at least in part, via miRNA-21, in hepatoma cells. To test this hypothesis, first HBx was over expressed in both Huh 7 and Hep G2 cells and the effect on cell proliferation was studied. In parallel experiments, as a positive control, eGFP-N1 plasmid vector was used to determine the transfection efficiency and was found to be more than 80% in all the experiments in both Huh 7 (Figure 1A and 1B) and Hep G2 cells (Figure 1C & 1D). HBx expression was assessed in HBx transfected cells and it was found that the HBx protein was expressed at high levels in both HBx-transfected Huh7 and Hep G2 cells (Figure 2A) but no expression was observed in control or empty vector-transfected cells. Previously it was shown that HBx was involved in the proliferation of HCC cells. Hence, the proliferation was analyzed in HBx over-expressing cells using WST1 assay as described in materials and methods. The proliferation of Huh 7 and Hep G2 cells transfected with HBx plasmid increased to 3.7 fold and 4.5 fold respectively (Figure 2B & 2C) compared to cells transfected with control plasmid (empty vector).

**Figure 3:**
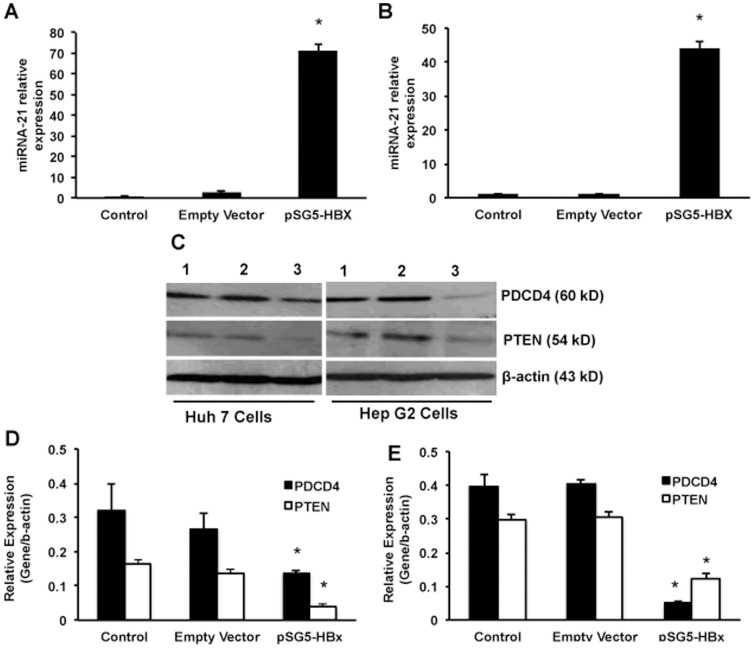
Effect of HBx on PDCD4 and PTEN. HBx protein was over-expressed in both Huh 7 (A) and Hep G2 (B) cells and the expression of miRNA-21 was estimated using real time PCR (n = 3; *p<0.001). HBx was over-expressed in both Huh 7 and Hep G2 cells and the target proteins for miRNA-21 were determined using Western blots. This picture is a representative of the three experiments (C). Lane 1, Control; lane 2, Empty vector; and lane 3, HBx transfected cells. β-actin was used as loading control. These Western blots were quantified using Li-COR's image studio lite software and the data are presented for Huh 7 (D) and Hep G2 (E) cells (n = 3; *p<0.05).

Next, the effect of HBx over-expression on the intracellular expression of miRNA-21 was studied in both these cells. The cells were transfected with HBx or empty vector and the cells were collected either for real time PCR or Western blots after 48 hours of transfection. The total RNA enriched with miRNAs was isolated, cDNA was synthesized and real time RT-PCR was performed for miRNA-21 expression. The results showed that there was a 24-fold and 36-fold increase in the expression of miRNA-21 in HBx over-expressing Huh7 and Hep G2 cells compared to empty vector-transfected cells respectively (Figure 3A and 3B). Previously it has been shown by several researchers that the major target proteins for miRNA-21 are PDCD4 and PTEN. Both these proteins are involved in regulating apoptosis. Western blots were performed in the cell lysates collected after the over-expression of HBx, empty vector or control cells. The results showed that there was a significant decrease in the expression of both PDCD4 and PTEN (Figure 3C). The Western blots were quantified and the results showed that both PDCD4 and PTEN were inhibited 2- and 3-fold in Huh 7 cells, and 8- and 3-fold in Hep G2 cells (p<0.05; Figure 3D and 3E).

**Figure 4:**
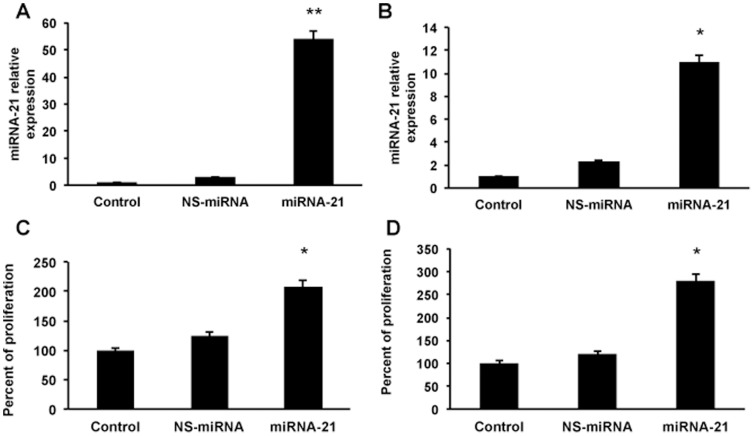
Role of miRNA-21 on proliferation of hepatoma cells. miRNA-21 was over-expressed in Hep G2 (A & C) and Huh 7 (B & D) cells. Relative expression of miRNA-21 was measured in the transfected cells (A & B) to determine the miRNA-21 levels (n = 3; *p<0.05 and **p<0.01). As an internal control, 5S RNA was quantified in all real time PCR experiments. Both C & D show the cell proliferation assays in miRNA-21 over-expressing cells. Cells were plated at a density of 8000/well in a 96-well plate for the proliferation assays. As a control in all these experiments NS-miRNA was used in parallel experiments (n = 3; *p<0.05).

**Figure 5:**
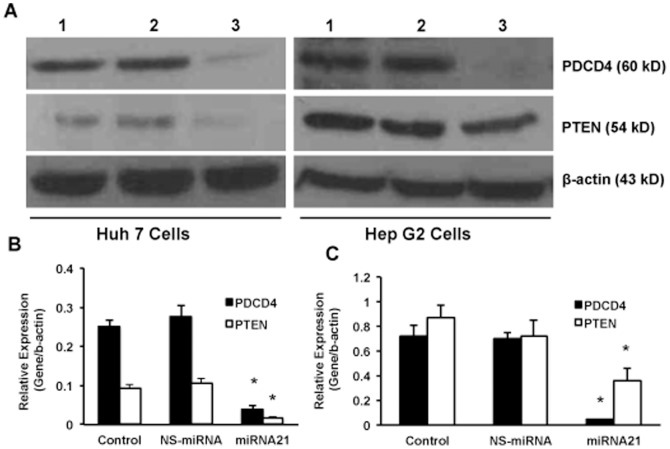
Effect of miRNA-21 on PDCD4 and PTEN proteins. A, shows the Western blot results of miRNA-21 target proteins, PDCD4 and PTEN in miRNA-21 over-expressing cells. As an internal control β-actin was used in all the Western blot experiments. This is a representative picture from three experiments. Lane 1, Control; lane 2, NS-miRNA transfected cells; and lane 3, miRNA-21 transfected cells. The Western blots were quantified and the data are presented for Huh 7 (B) and Hep G2 (C) cells (n = 3; *p<0.05).

Several studies have reported that miRNA-21 is up regulated in cancer tissues and their over-expression resulted in increased cell proliferation [7], [13], [24]. Effect of over-expression of miRNA-21 on the proliferation of the hepatoma cells was analyzed. For this, the cells were transfected with premiR-21 oligos using NeoFX siPORT transfection agent as described in materials and methods. There was a 17- and 5-fold increase in the intracellular levels of miRNA-21 (Figure 4A & 4B) compared to the NS-miRNA transfected cells of Huh 7 and Hep G2 respectively. Overexpression of miRNA-21 led to 2- and 2.3-fold increase in the proliferation of both Huh 7 and Hep G2 cells respectively (n = 3; p<0.05; Figure 4C & 4D). It was expected that when the proliferation goes up, miRNA-21 would inhibit its target proteins. After 72 hours of transfection, the cells were collected, protein was isolated and Western blots were performed for PDCD4 and PTEN. In all these experiments β-actin was used as loading control. The results showed that miRNA-21 transfected cells showed a significant decrease in the expression of these two proteins (Figure 5A). These Western blot images were quantified using Li-COR's image studio lite software from 3 experiments and the data showed that there was a significant decrease in these target proteins (n = 3; p<0.01). These results clearly show that HBx induces proliferation, at least in part, via inducing miRNA-21.

**Figure 6:**
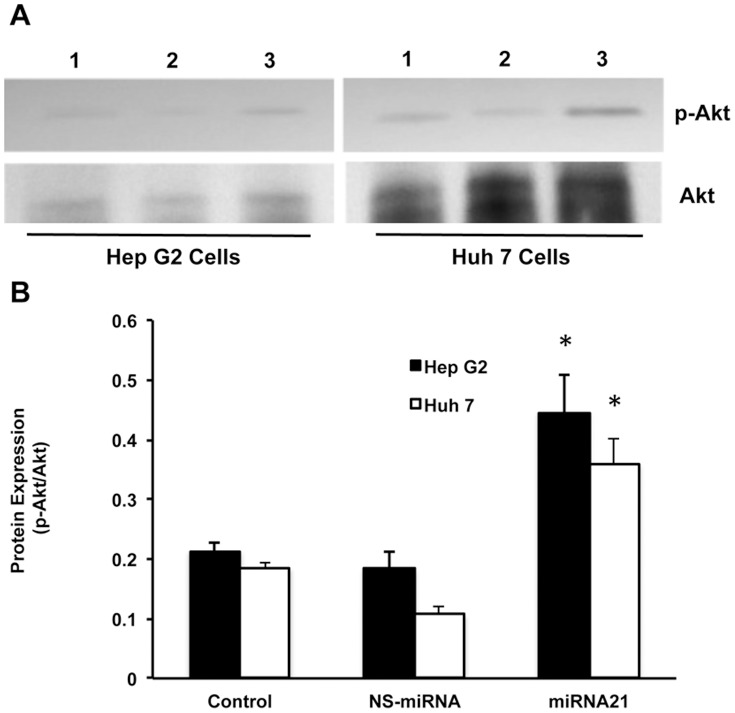
Effect of miRNA-21 on phospho-akt levels. A, shows the Western blot results of phsopho-akt and akt proteins in the miRNA-21-transfected cells. This is a representative picture from three experiments. Lane 1, Control; lane 2, NS-miRNA transfected cells; and lane 3, miRNA-21 transfected cells. B, Western blots were quantified and the data are presented in the graph (n = 3; *p<0.05).

**Figure 7:**
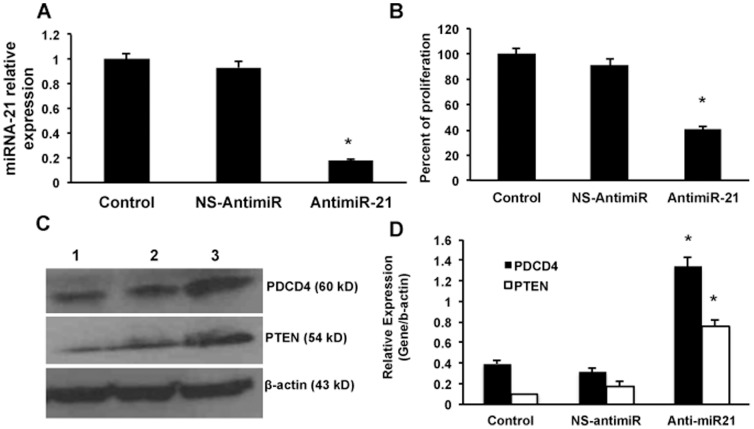
Effect of anti-miR21 on proliferation and miRNA-21 target proteins. Intracellular miRNA-21 was inhibited using anti-miR-21 oligos in Hep G 2.2.1.5 cells. A, Relative expression of miRNA-21 was measured in the anti-miR-21 transfected cells and NS-anti-miR was used as control in all the experiments (n = 3; *p<0.01). B, The proliferation assay was also performed in anti-miR-21 transfected cells (n = 3; *p<0.05). C, Western blot was performed to measure the protein levels of miRNA-21 target proteins, PDCD4 and PTEN in anti-miR-21 transfected cells. As an internal control β-actin was used in all the Western blot experiments. Lane 1, Control; lane 2, NS-anti-miR transfected cells; and lane 3, anti-miR-21 transfected cells. D, The Western blots were quantified and the data are presented from three experiments (*p<0.05).

Next, we wanted to analyze the downstream signaling pathway. The cellular protein was isolated from the miRNA-21 transfected cells and Western blotting was performed for phospho-Akt and Akt. As expected the overexpression of miRNA-21 resulted in the activation of phospho-Akt to two-fold levels compared to the control or the NS-miRNA transfected cells (Figure 6A and 6B; n = 3; p<0.05). Since over-expressing miRNA-21 results in decreased proliferation, we anticipated that inhibiting miRNA-21 would increase its target proteins and decrease cell proliferation. To further confirm this hypothesis, HBV stably transfected cell line, Hep G 2.2.1.5 cells, were used for inhibiting intracellular miRNA-21 levels by anti-miR-21 oligos and the cell proliferation and target protein expression were analyzed. As a control non-specific anti-miRNA oligos (NS-antimiR) were used in all the miRNA-21 inhibitor experiments. The intracellular expression of miRNA-21 was studied in anti-miR-21 transfected cells using real time PCR experiments. It was found that there was an 80% inhibition of the intracellular levels of miRNA-21 (Figure 7A). Also a significant inhibition of proliferation of Hep G 2.2.1.5 was seen after transfection with anti-miR-21 (n = 3; p<0.05; Figure 7B). Next, target proteins of miRNA-21 were studied and as expected it was found that there was a significant increase in the levels of both PDCD4 and PTEN upon inhibition of miRNA-21 (Figure 7C). Figure 7D shows the quantitative Western blot results from 3 experiments (p<0.05).

**Figure 8:**
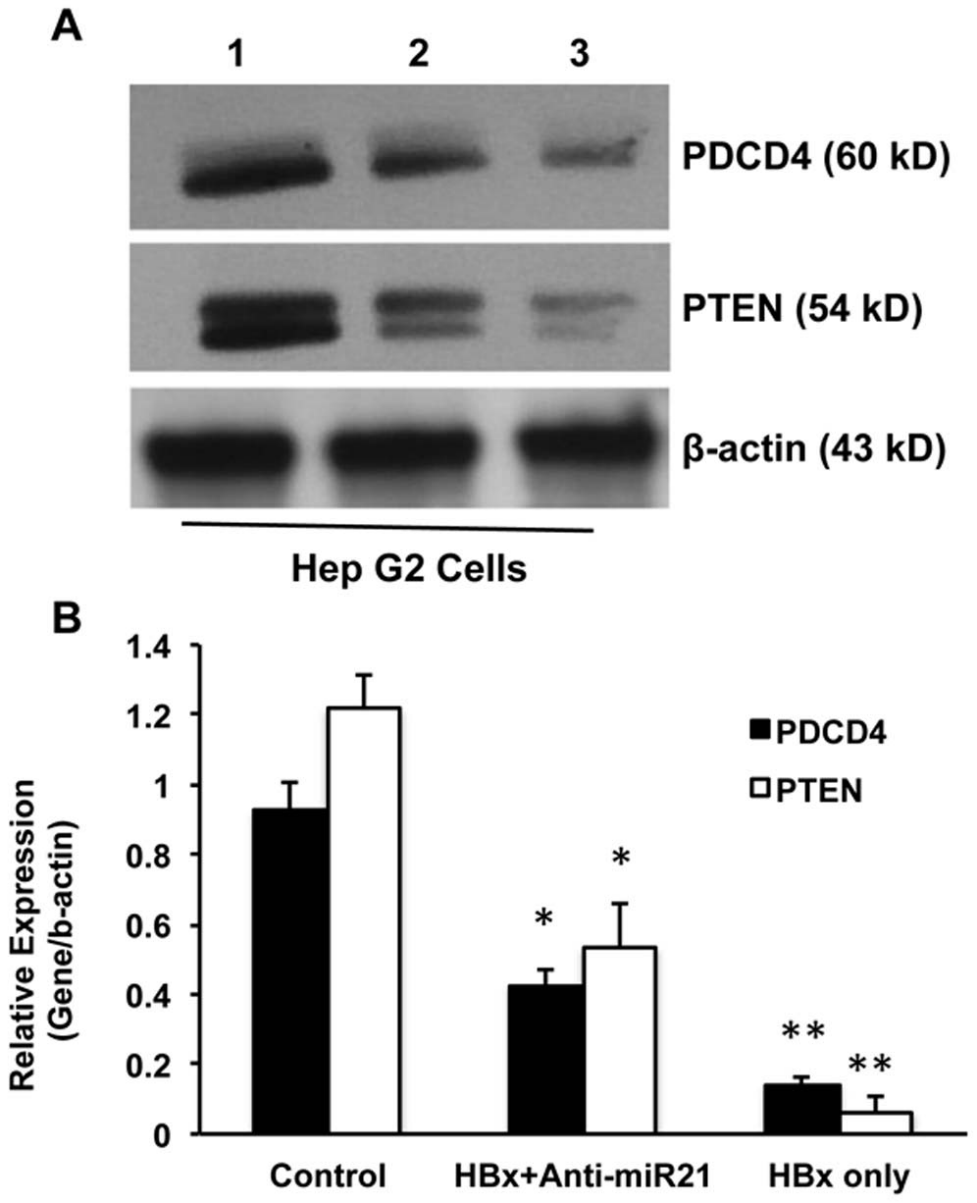
Effect of HBx in miRNA-21-induced PDCD4 and PTEN in Hep G2 cells. Hep G2 cells were transfected with anti-miR21, followed by transfection with HBx plasmid. After 48 hours of HBx plasmid transfection, the cells were collected and Western blots for PDCD4 and PTEN were performed. A, shows the protein levels of miRNA-21 target proteins, PDCD4 and PTEN in anti-miR-21 transfected cells. As an internal control β-actin was used in all the Western blot experiments. Lane 1, Control; lane 2, Anti-miR21 and HBx-transfected cells; and lane 3, HBx only transfected cells. B, The Western blots were quantified and the data are presented from three experiments. (*p<0.05, compared with HBx only transfected cell; **p<0.01, compared with control cells).

**Figure 9:**
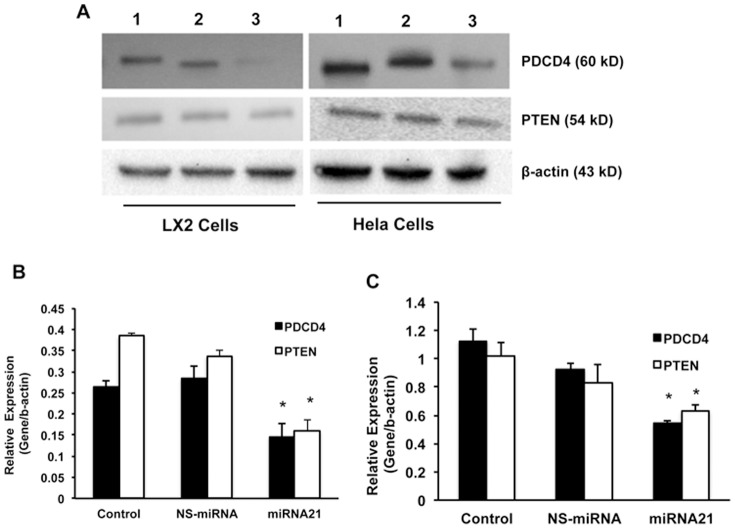
Effect of miRNA-21 on PDCD4 and PTEN proteins in LX2 and Hela Cells. A, shows a representative picture of Western blot results of miRNA-21 target proteins, PDCD4 and PTEN in miRNA-21 over-expressing LX2 and Hela cells. As an internal control β-actin was used in all the Western blot experiments. Lane 1, Control; lane 2, NS-miRNA transfected cells; and lane 3, miRNA-21 transfected cells. The Western blots were quantified and the data are presented for LX2 (B) and Hela (C) cells (n = 3; *p<0.05).

Next, the role of miRNA-21 in HBx-induced cellular proliferation was studied. For this, the cells were transfected with anti-miR-21 and then the same cells were transfected with HBx plasmid. The cells were collected after 48 hours of HBx plasmid transfection and the cellular protein was isolated. Western blotting was performed for PDCD4 and PTEN (Figure 8A) and it was found that transfection with HBx alone resulted in maximum inhibition of PDCD4 and PTEN compared to control cells (Figure 8B; 90% and 80% inhibition respectively; n = 3; p<0.01), while inhibition of miRNA-21 partially recovered the expression of both these proteins (Figure 8B; 45% and 50% inhibition respectively compared to the control cells; n = 3; p<0.01). Finally, the effect of miRNA-21 was checked in other cell lines, LX2 (hepatic stellate cell line) cells and Hela (cervical cancer cell line) cells. The results showed that both PDCD4 and PTEN were inhibited just like hepatic cancer cell lines (Figure 9A). The blots were quantitated and the quantitative data showed that both PDCD4 and PTEN were significantly inhibited in both LX2 cells (Figure 9B) and Hela cells (Figure 9C).

## Discussion

Previously we have shown that miRNA-21 was up-regulated when there was liver regeneration after injury [31] or after partial hepatectomy [33]. Reports from other investigators have shown that miRNA-21 is involved in enhancing cell proliferation and is up-regulated in several cancer tissues [7], [13], [24]. Studies have shown that HBx plays an important role in the progression of HBV-associated HCC [2], [34]. HBx has been shown to induce various signaling pathways and cellular proteins that could link HCC with HBV infection [7]–[11]. Till date, very little is known on the role of miRNAs in HBx-induced proliferation and metastasis. In this study, we have delineated the role of HBx on the expression of miRNA-21 expression and its role in inducing the proliferation of hepatoma cells.

It was shown that HBx inhibited apoptosis via activation of the Phosphatidyl inositol 3-kinase (PI3K) pathway and inhibition of the PI3K pathway blocked the anti-apoptotic effect of HBx [9], [35]. A similar study in Hep3B cells that were stably transfected with HBx showed that HBx also inhibited TGF-β-induced DNA fragmentation through a PI3K-dependent pathway [36]. In another study, HBx was shown to activate the PI3K pathway and inhibit apoptosis by down regulating the expression of PTEN in Chang cells [37]. Our data show that PTEN is inhibited by the over-expression of HBx in both Hep G2 and Huh7 cells. Our data also showed that PDCD4 was inhibited when HBx was over-expressed. Since PDCD4 and PTEN are pro-apoptotic proteins, our data is in agreement with the previous data that HBx inhibits apoptosis and enhances cellular proliferation in hepatoma cells [26], [28].

Emerging evidences suggest that HBx modulates the miRNA in HCC. Previous studies have shown that miRNA-21 is elevated in various cancer tissues and it promotes cell proliferation of various cancerous cells. However, the relationship between HBx and miRNA-21 expression was not known. To analyze this, we hypothesized that HBx could induce the expression of miRNA-21, which in turn could induce the cell proliferation via inhibiting the pro-apoptotic proteins, PDCD4 and PTEN, target genes of miRNA-21. It was shown that HBx upregulates miRNA-29a, which in turn inhibits PTEN in hepatoma cells, leading to increased migration [18]. Our data also show that over-expression of HBx inhibited PTEN in our cell culture model system. Indeed, over-expression of HBx in hepatoma cells not only increased cell proliferation but also induced the intracellular expression of miRNA-21. This data shows that HBx induces cell proliferation, at least in part, via inducing miRNA-21 in HBV associated HCC. Previous studies have shown that miRNAs play an important role in HBx-induced cell proliferation. HBx was shown to inhibit the expression of miRNA-148a to induce tumorigenesis [14]. Expression of miRNA-148a inhibited mTOR via inhibition of the expression of Akt and Erk proteins [14]. Our study confirms the previous finding that HBx utilizes miRNAs for its tumorigenesis. Our data show that HBx enhances the expression of miRNA-21 and increases Akt by inhibiting PTEN, thereby increasing the proliferation of hepatic cells.

When miRNA-21 was over expressed in Hep G2 and Huh 7 cells, cell proliferation was enhanced. This is in agreement with several studies, which show that miRNA-21 increases cell proliferation [24], [26], [28]. It was studied whether over expressing miRNA-21 could inhibit its target proteins, PDCD4 and PTEN. The resulting inhibition of PDCD4 and PTEN showed that the exogenously transfected pre-miRNA-21 were functionally active. Other studies also have shown that miRNA-21 is involved in proliferation via enhancing PI3K pathway [9], [35], [36]. Previously, Kong et al [18] have shown that HBx induces the expression of miRNA-29a, and induces migration of Hep G2-X cells (HBx-transfected hepatoma cells) while inhibition of miRNA-29a resulted in a complete abolition of migration of these cells. In our study, we found that HBx induces cellular proliferation, at least in part, via miRNA-21 in Hep G2 cells. These data suggest that HBx uses more than one mechanism to induce the cell proliferation [18].

It has been reported that HBx up-regulated miR-143 through NF-κB, promoting HCC metastasis in an athymic nude mouse model and miR-29a promoted migration of HepG2 cells via HBx targeting PTEN [18], [21]. Here, we show that when HBx is ectopically expressed in hepatoma cells, it up-regulated miRNA-21 significantly, which caused inhibition of its target proteins, PDCD4 and PTEN. Both PDCD4 and PTEN contain putative binding sites for miRNA-21 in their 3′UTRs.

Onco-protein HBx, a transcriptional transactivator encoded by the hepatitis B virus has been widely accepted to create a pro-proliferative environment in the human hepatocytes by activating various cell growth-promoting signaling pathways as well as deregulating cell cycle control genes which ultimately augments neoplastic transformation [34]. HBx protein induced proliferation of HepG2 and Huh7 hepatoma cells by enhancing the expression of miRNA-21, indicating that oncomiR-21, which is reported to be up-regulated in HCC, could possibly get up-regulated much before the hepatocytes becomes malignant.

When anti-miR-21 was transfected in HepG2.2.15 cells in which the Hepatitis B virus is stably integrated, the proliferation was significantly inhibited and the intracellular expression of miRNA-21 was also down regulated.

**Figure 10:**
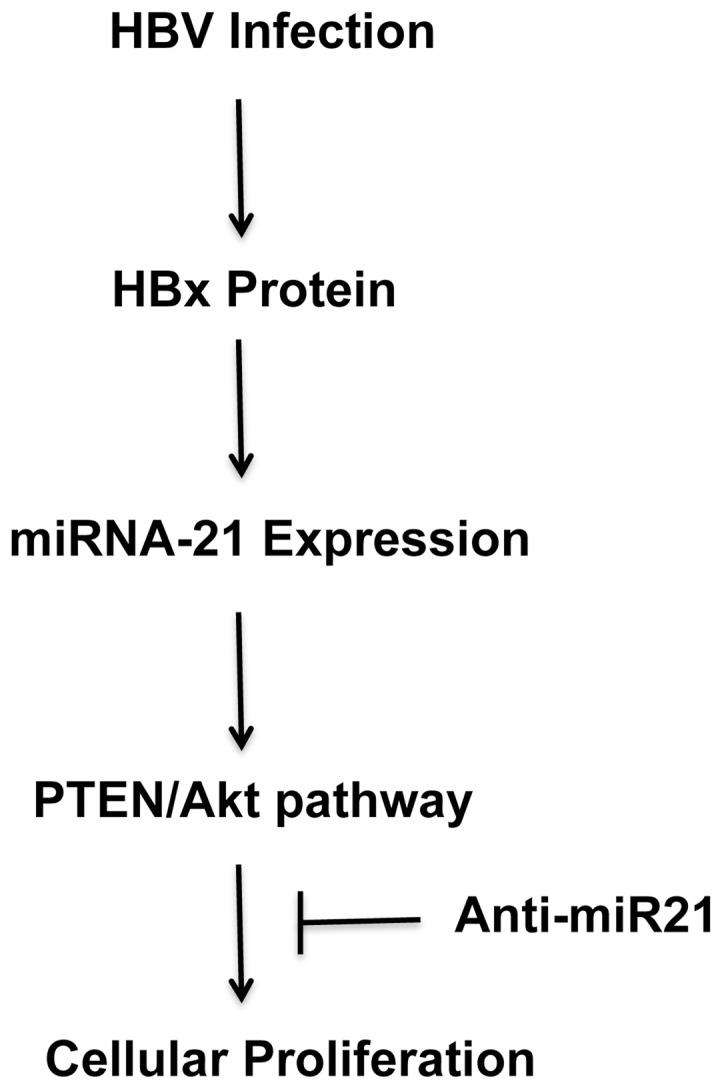
Proposed Model of HBx-miRNA-21 relationship. Schematic diagram showing the relationship between HBx, miRNA-21, akt and cellular proliferation in hepatic cells.

In summary, our data show that HBx at least in part, induces cell proliferation via inducing miRNA-21, which in turn inhibits PDCD4 and PTEN, and activates Akt. The proposed model is presented in Figure 10. Identifying key miRNAs, which are modulated at early stages of cancer, is important for novel therapeutic interventions that could prevent further disease progression. Nonetheless, further studies are required to confirm these findings using in vivo expression studies.

## References

[1] El-Serag HB (2011) Hepatocellular carcinoma. N Engl J Med 365: 1118–1127. 21992124 10.1056/NEJMra1001683

[2] Liang TJ (2009) Hepatitis B: the virus and disease. Hepatology 49: S13–21. 19399811 10.1002/hep.22881 PMC2809016

[3] Cha MY, Ryu DK, Jung HS, Chang HE, Ryu WS (2009) Stimulation of hepatitis B virus genome replication by HBx is linked to both nuclear and cytoplasmic HBx expression. J Gen Virol 90: 978–986. 19264639 10.1099/vir.0.009928-0

[4] Yang B, Bouchard MJ (2012) The hepatitis B virus X protein elevates cytosolic calcium signals by modulating mitochondrial calcium uptake. J Virol 86: 313–327. 22031934 10.1128/JVI.06442-11 PMC3255901

[5] Paterlini P, Poussin K, Kew M, Franco D, Brechot C (1995) Selective accumulation of the X transcript of hepatitis B virus in patients negative for hepatitis B surface antigen with hepatocellular carcinoma. Hepatology 21: 313–321. 7843699

[6] Wang Y, Cui F, Lv Y, Li C, Xu X, et al (2004) HBsAg and HBx knocked into the p21 locus causes hepatocellular carcinoma in mice. Hepatology 39: 318–324. 14767984 10.1002/hep.20076

[7] Kong GY, Zhang JP, Zhang S, Shan CL, Ye LH, et al (2011) Hepatitis B virus X protein promotes hepatoma cell proliferation via upregulation of MEKK2. Acta Pharmacol Sin 32: 1173–1180. 21804577 10.1038/aps.2011.52 PMC4003298

[8] Xia LM, Huang WJ, Wu JG, Yang YB, Zhang Q, et al (2009) HBx protein induces expression of MIG and increases migration of leukocytes through activation of NF-kappaB. Virology 385: 335–342. 19157479 10.1016/j.virol.2008.11.042

[9] Chung TW, Lee YC, Kim CH (2004) Hepatitis B viral HBx induces matrix metalloproteinase-9 gene expression through activation of ERK and PI-3K/AKT pathways: involvement of invasive potential. FASEB J 18: 1123–1125. 15132991 10.1096/fj.03-1429fje

[10] Huang WC, Chen WS, Chen YJ, Wang LY, Hsu SC, et al (2012) Hepatitis B virus X protein induces IKKalpha nuclear translocation via Akt-dependent phosphorylation to promote the motility of hepatocarcinoma cells. J Cell Physiol 227: 1446–1454. 21618535 10.1002/jcp.22860

[11] Zhang F, Wang Q, Ye L, Feng Y, Zhang X (2010) Hepatitis B virus X protein upregulates expression of calpain small subunit 1 via nuclear factor-kappaB/p65 in hepatoma cells. J Med Virol 82: 920–928. 20419804 10.1002/jmv.21753

[12] Lara-Pezzi E, Majano PL, Yanez-Mo M, Gomez-Gonzalo M, Carretero M, et al (2001) Effect of the hepatitis B virus HBx protein on integrin-mediated adhesion to and migration on extracellular matrix. J Hepatol 34: 409–415. 11322202 10.1016/s0168-8278(00)00090-8

[13] Gearhart TL, Bouchard MJ (2011) The hepatitis B virus HBx protein modulates cell cycle regulatory proteins in cultured primary human hepatocytes. Virus Res 155: 363–367. 20934470 10.1016/j.virusres.2010.09.023 PMC3010534

[14] Xu X, Fan Z, Kang L, Han J, Jiang C, et al (2013) Hepatitis B virus X protein represses miRNA-148a to enhance tumorigenesis. J Clin Invest 123: 630–645. 23321675 10.1172/JCI64265 PMC3561812

[15] Wei X, Xiang T, Ren G, Tan C, Liu R, et al (2013) miR-101 is down-regulated by the hepatitis B virus x protein and induces aberrant DNA methylation by targeting DNA methyltransferase 3A. Cell Signal 25: 439–446. 23124077 10.1016/j.cellsig.2012.10.013

[16] Yuan K, Lian Z, Sun B, Clayton MM, Ng IO, et al (2012) Role of miR-148a in hepatitis B associated hepatocellular carcinoma. PLoS One 7: e35331. 22496917 10.1371/journal.pone.0035331 PMC3322146

[17] Wu G, Yu F, Xiao Z, Xu K, Xu J, et al (2011) Hepatitis B virus X protein downregulates expression of the miR-16 family in malignant hepatocytes in vitro. Br J Cancer 105: 146–153. 21629246 10.1038/bjc.2011.190 PMC3137408

[18] Kong G, Zhang J, Zhang S, Shan C, Ye L, et al (2011) Upregulated microRNA-29a by hepatitis B virus X protein enhances hepatoma cell migration by targeting PTEN in cell culture model. PLoS One 6: e19518. 21573166 10.1371/journal.pone.0019518 PMC3088678

[19] Varnholt H (2008) The role of microRNAs in primary liver cancer. Ann Hepatol 7: 104–113. 18626426

[20] Ura S, Honda M, Yamashita T, Ueda T, Takatori H, et al (2009) Differential microRNA expression between hepatitis B and hepatitis C leading disease progression to hepatocellular carcinoma. Hepatology 49: 1098–1112. 19173277 10.1002/hep.22749

[21] Zhang X, Liu S, Hu T, Liu S, He Y, et al (2009) Up-regulated microRNA-143 transcribed by nuclear factor kappa B enhances hepatocarcinoma metastasis by repressing fibronectin expression. Hepatology 50: 490–499. 19472311 10.1002/hep.23008

[22] Krichevsky AM, Gabriely G (2009) miR-21: a small multi-faceted RNA. J Cell Mol Med 13: 39–53. 19175699 10.1111/j.1582-4934.2008.00556.x PMC3823035

[23] Talotta F, Cimmino A, Matarazzo MR, Casalino L, De Vita G, et al (2009) An autoregulatory loop mediated by miR-21 and PDCD4 controls the AP-1 activity in RAS transformation. Oncogene 28: 73–84. 18850008 10.1038/onc.2008.370

[24] Frezzetti D, De Menna M, Zoppoli P, Guerra C, Ferraro A, et al (2011) Upregulation of miR-21 by Ras in vivo and its role in tumor growth. Oncogene 30: 275–286. 20956945 10.1038/onc.2010.416

[25] Yang X, Wang J, Guo SL, Fan KJ, Li J, et al (2011) miR-21 promotes keratinocyte migration and re-epithelialization during wound healing. Int J Biol Sci 7: 685–690. 21647251 10.7150/ijbs.7.685 PMC3107477

[26] Gaur AB, Holbeck SL, Colburn NH, Israel MA (2011) Downregulation of Pdcd4 by mir-21 facilitates glioblastoma proliferation in vivo. Neuro Oncol 13: 580–590. 21636706 10.1093/neuonc/nor033 PMC3107097

[27] Zhou X, Ren Y, Moore L, Mei M, You Y, et al (2010) Downregulation of miR-21 inhibits EGFR pathway and suppresses the growth of human glioblastoma cells independent of PTEN status. Lab Invest 90: 144–155. 20048743 10.1038/labinvest.2009.126

[28] Meng F, Henson R, Wehbe-Janek H, Ghoshal K, Jacob ST, et al (2007) MicroRNA-21 regulates expression of the PTEN tumor suppressor gene in human hepatocellular cancer. Gastroenterology 133: 647–658. 17681183 10.1053/j.gastro.2007.05.022 PMC4285346

[29] Zhang S, Li J, Jiang Y, Xu Y, Qin C (2009) Programmed cell death 4 (PDCD4) suppresses metastastic potential of human hepatocellular carcinoma cells. J Exp Clin Cancer Res 28: 71. 19480673 10.1186/1756-9966-28-71 PMC2705348

[30] Venugopal SK, Jiang J, Kim TH, Li Y, Wang SS, et al (2010) Liver fibrosis causes downregulation of miRNA-150 and miRNA-194 in hepatic stellate cells, and their overexpression causes decreased stellate cell activation. Am J Physiol Gastrointest Liver Physiol 298: G101–106. 19892940 10.1152/ajpgi.00220.2009 PMC2806096

[31] Yoon S, Kim TH, Natarajan A, Wang SS, Choi J, et al (2010) Acute liver injury upregulates microRNA-491-5p in mice, and its overexpression sensitizes Hep G2 cells for tumour necrosis factor-alpha-induced apoptosis. Liver Int 30: 376–387. 20015148 10.1111/j.1478-3231.2009.02181.x

[32] Pfaffl MW (2001) A new mathematical model for relative quantification in real-time RT-PCR. Nucleic Acids Res 29: e45. 11328886 10.1093/nar/29.9.e45 PMC55695

[33] Chen X, Murad M, Cui YY, Yao LJ, Venugopal SK, et al (2011) miRNA regulation of liver growth after 50% partial hepatectomy and small size grafts in rats. Transplantation 91: 293–299. 21183868 10.1097/TP.0b013e318204756c

[34] Benhenda S, Cougot D, Buendia MA, Neuveut C (2009) Hepatitis B virus X protein molecular functions and its role in virus life cycle and pathogenesis. Adv Cancer Res 103: 75–109. 19854353 10.1016/S0065-230X(09)03004-8

[35] Lee YI, Kang-Park S, Do SI, Lee YI (2001) The hepatitis B virus-X protein activates a phosphatidylinositol 3-kinase-dependent survival signaling cascade. J Biol Chem 276: 16969–16977. 11278872 10.1074/jbc.M011263200

[36] Shih WL, Kuo ML, Chuang SE, Cheng AL, Doong SL (2000) Hepatitis B virus X protein inhibits transforming growth factor-beta -induced apoptosis through the activation of phosphatidylinositol 3-kinase pathway. J Biol Chem 275: 25858–25864. 10835427 10.1074/jbc.M003578200

[37] Chung TW, Lee YC, Ko JH, Kim CH (2003) Hepatitis B Virus X protein modulates the expression of PTEN by inhibiting the function of p53, a transcriptional activator in liver cells. Cancer Res 63: 3453–3458. 12839924

